# Glomerular disease associated with cancer: a case series of paraneoplastic nephrotic syndrome

**DOI:** 10.3389/fmed.2025.1609192

**Published:** 2025-10-30

**Authors:** Mengsi Hu, Qiqi Ma, Liguang Wang, Tingwei Zhang, Jiangong Lin, Xiaowei Yang, Zhimei Lv, Rong Wang

**Affiliations:** ^1^Department of Nephrology, Shandong Provincial Hospital Affiliated to Shandong First Medical University, Jinan, China; ^2^Department of Nephrology, Shandong Provincial Hospital, Cheeloo College of Medicine, Shandong University, Jinan, China; ^3^Department of Nephrology, Jiangxi Medical College, The Second Affiliated Hospital, Nanchang University, Nanchang, China; ^4^Department of Rheumatology and Immunology, The Second Hospital of Shandong University, Jinan, China; ^5^Department of Minimally Invasive Comprehensive Treatment of Cancer, Shandong Provincial Hospital Affiliated to Shandong First Medical University, Jinan, China; ^6^Department of Nephrology, The Affiliated Taian City Central Hospital of Qingdao University, Taian, China

**Keywords:** paraneoplastic glomerular disease, ovarian cancer, pancreatic neuroendocrine tumor, bladder cancer, paraneoplastic nephrotic syndrome

## Abstract

The association between glomerular diseases and malignancies has been recognized since the 1920s. A diverse spectrum of glomerular lesions can occur in various neoplasms, including both hematologic malignancies and solid tumors. This study presents a case series of paraneoplastic nephrotic syndrome (PNS) associated with three solid tumors: ovarian cancer, pancreatic neuroendocrine tumor (NET), and bladder cancer. The occurrence of PNS is rarely reported in association with these malignancies. Notably, all patients achieved complete or partial remission without receiving corticosteroids or immunosuppressant therapy. These observations accentuate the critical role of malignancy in the pathogenesis of glomerulopathy and underscore the therapeutic primacy of oncological control in such patients.

## Introduction

The association between glomerular diseases and malignancies was first recognized as early as the 1920s by Galloway in patients with Hodgkin’s disease (1). Subsequent studies have reported that renal involvement may occur in over 7% of cancer patients. (2, 3). A recent Brazilian retrospective cohort analysis found that 1.97% of patients had concurrent glomerulopathy and neoplasms, with hematologic malignancies being the most common (35.8%), followed by colon and gynecologic tumors (4).

In this study, we present a case series of paraneoplastic nephrotic syndrome (PNS) in association with three solid tumors: ovarian cancer, pancreatic neuroendocrine tumor (NET), and bladder cancer. The occurrence of PNS in conjunction with these malignancies is rarely documented. Notably, all patients achieved complete or partial remission without corticosteroid or immunosuppressive therapy. These observations accentuate the critical role of malignancy in the pathogenesis of glomerulopathy and underscore the therapeutic primacy of oncological control in these patients.

## Case report

### Case 1

A 71-year-old Chinese woman presented with bilateral lower limb edema. Her medical history included well-controlled diabetes mellitus and hypertension, managed with irbesartan. Physical examination revealed periorbital and lower extremity edema with normal blood pressure. Laboratory tests demonstrated nephrotic-range proteinuria (24 h UTP 12.80 g/d), severe hypoalbuminemia (serum albumin 19.30 g/L), and marked hyperlipidemia (triglycerides 4.56 mmol/L, total cholesterol 8.83 mmol/L, and LDL-C 5.73 mmol/L). Renal function remained relatively normal (serum creatinine 0.70 mg/dL, eGFR 87.40 mL/min/1.73m^2^, CKD-EPI 2009). Serological tests, including phospholipase A2 receptor antibodies (PLA2R-Ab), autoantibodies, viral markers, serum complement, and immunofixation electrophoresis, were all negative. Fundoscopy showed no diabetic retinopathy, consistent with her glycemic control (HbA1c 6.1%).

**Figure 1:**
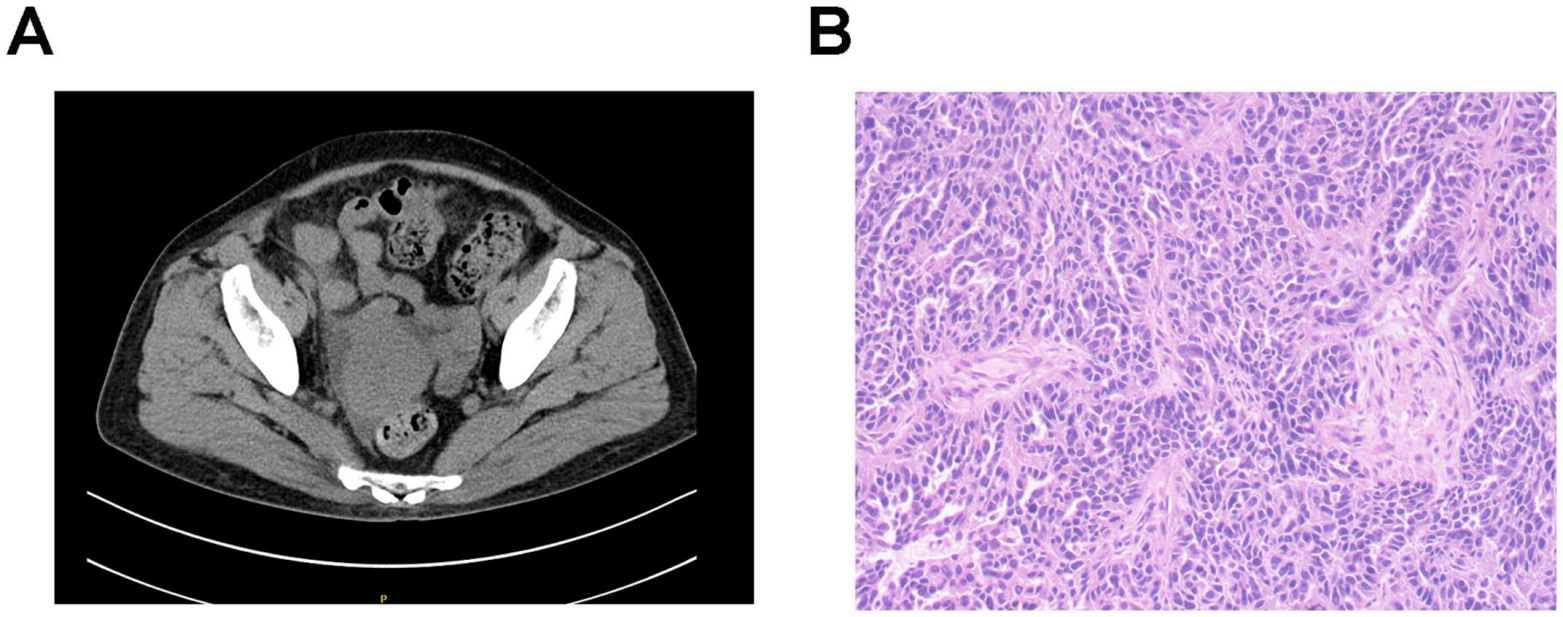
Imaging and pathological characteristics of ovarian cancer in patient 1. **(A)** Abdominal CT scanning indicates an irregular soft tissue near the uterus without obvious boundaries with the uterus. **(B)** Histopathology shows right adnexal high-grade serous carcinoma. Tumor dimensions: 6.5 × 5 × 4 cm. Lymphovascular space invasion present (cancer emboli identified). Metastatic lesions identified in: right fallopian tube, right parietal peritoneum, and omentum. Uterus and left adnexa: carcinomatous involvement of the uterine serosal surface, and the left ovary and left fallopian tube show no significant lesions. Immunohistochemistry results: WT-1 (+), Vimentin (−), P16 + (patchy/mottled staining pattern), P53 (−), Ki-67 + (60% proliferation index), ER (−), PR (−), and PAX8 (+).

Gynecological ultrasound and abdominal CT scanning revealed a right adnexal cystic-solid mass (9.1 × 7.4 × 6.2 cm) and an irregular peri-uterine soft tissue lesion with poorly defined boundaries (5.3 × 6.0 cm), respectively. Tumor markers were significantly elevated (CA125 311.00 U/mL [normal<25], HE4 1414.00 pmol/L [normal<140]). The patient declined a renal biopsy and underwent cytoreductive surgery, which confirmed stage IIIC high-grade serous ovarian carcinoma (Figure 1). Post-operative management included intraperitoneal carboplatin and supportive therapy (metformin, insulin, statins, irbesartan, low molecular weight heparin, and diuretics). Tumor markers significantly declined post-operatively (CA125 191 U/mL, HE4 145 pmol/L), while proteinuria persisted (3+), and serum albumin decreased to 16 g/L but increased to 25.9 g/L after albumin infusions. Her renal function remained stable throughout hospitalization, and the patient was discharged with persistent edema.

During the 6-month follow-up, the patient received paclitaxel and carboplatin-based adjuvant chemotherapy. Notably, 1 month after surgery and prior to initiating chemotherapy, laboratory tests showed signs of NS remission, evidenced by reduced proteinuria (2+), improved serum albumin (30 g/L), and resolved edema, without albumin infusion, steroids, or immunosuppressants. Her tumor markers continued to decline at this time point (CA125 45.3 U/mL, HE4 89.0 pmol/L). Complete remission of NS was observed at 4-month post-surgery, with sustained normalization of both CA125 and HE4 levels. At 1-year follow-up, CT scanning showed disease progression with new soft tissue lesions in the left pelvic wall. Consequently, over the next 6 months, the patient received six cycles of combined paclitaxel/carboplatin chemotherapy, with bevacizumab added in the first and the last cycles, and her tumor markers increased (CA125 24.8 U/mL, HE4 299 pmol/L). Nevertheless, despite tumor progression, renal remission persisted throughout the subsequent 1.5 years, with no proteinuria (24 h UTP 0.09 g/d), normal serum albumin (38.2 g/L), and stable renal function (eGFR 90.0 mL/min/1.73m^2^).

### Case 2

**Figure 2:**
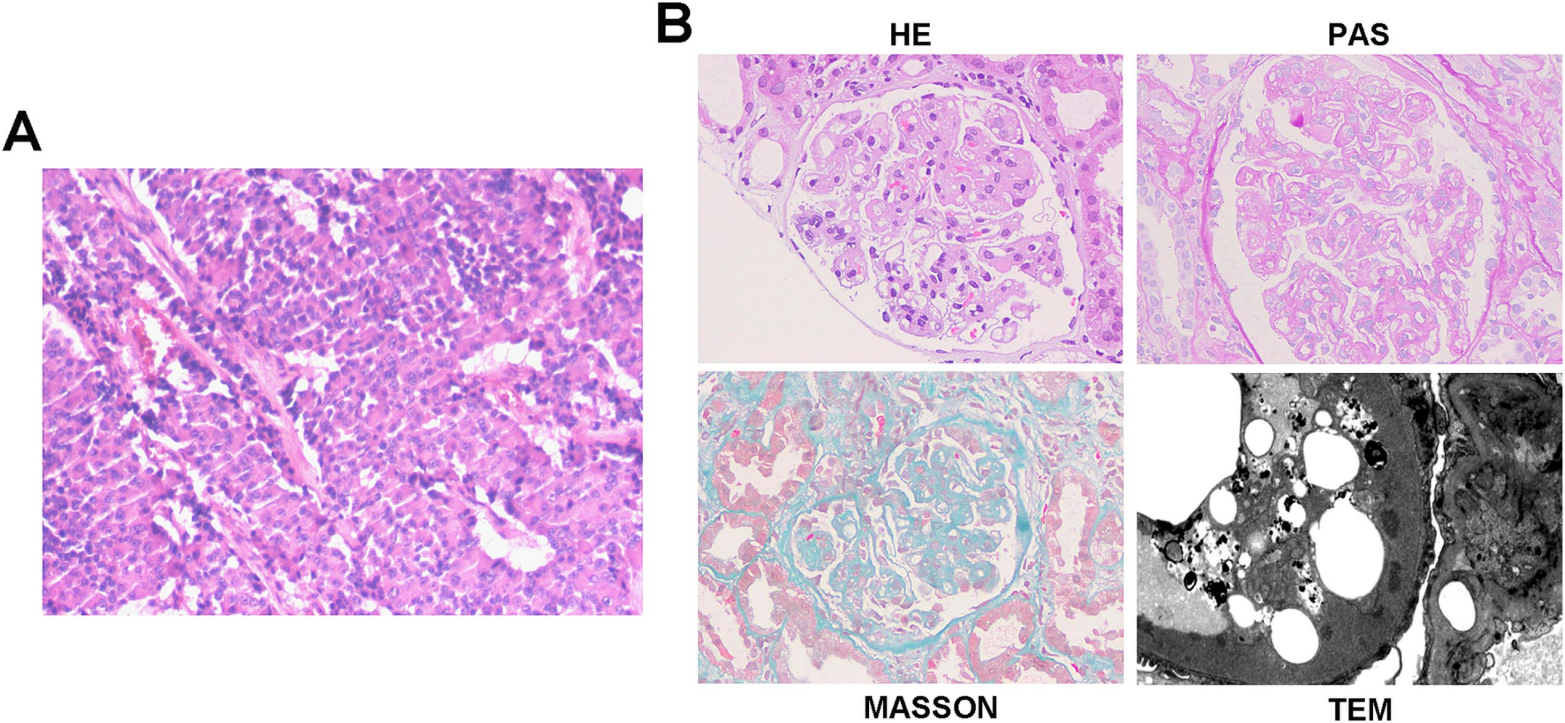
Histopathology of pancreatic NET and kidney in case 2. **(A)** Histopathology shows pancreatic neuroendocrine tumor, grade 2 (NET G2). Tumor size: 11 × 6 cm. Mitotic rate: 5 per 50 high-power fields (HPFs). Status of resection margins: Negative for tumor involvement. Adjacent structures: pancreatic transection margin: tumor-free; splenic parenchyma: no tumor identified; left adrenal gland: uninvolved. Hepatic metastases: left hepatic lobe: ingle metastatic deposit (10 × 9 cm) confirmed as metastatic NET; resection margin: clear; right hepatic lobe: two metastatic foci (4.5 × 3.5 cm and 2 × 1.8 cm), histologically consistent with metastatic NET; larger lesion abuts the capsular surface; resection margins: free of tumor. Lymph node status: peripancreatic lymph node (1/1): no metastatic involvement. Immunohistochemistry results: CK(AE1/AE3) (+), Vimentin (−), CK7(−), CK8/18(+), CK19 (+), CD56 (+), Syn (+), CgA (+), B-Catenin (+), and Ki-67(20%). **(B)** Representative of renal pathology of case 2.

A 57-year-old Chinese man was admitted with bilateral lower limb edema for 2 months and elevated serum creatinine for 20 days. His medical history included hypertension, treated with calcium channel blockers, and a pancreatic NET G2 (Figure 2A), for which he had undergone distal pancreatectomy, splenectomy, and metastatic liver resection 18 months prior. Physical examination showed lower extremity edema. Laboratory tests revealed nephrotic-range proteinuria (24 h UTP 4.98 g/d), microscopic hematuria (17.2 RBCs/HPF), hypoalbuminemia (serum albumin 24.9 g/L), hyperlipidemia, and preserved renal function (eGFR 85.0 mL/min·1.73m^2^, CKD-EPI 2009). All serological tests were negative.

Renal biopsy showed 29 glomeruli, eight with segmental sclerosis and adhesions, diffuse capillary wall thickening with double contours, mild-to-moderate mesangial proliferation, and scattered deposits on Masson staining. Interstitial fibrosis (10–20%) and tubular atrophy (20%) with inflammation were present. A diagnosis of mesangial proliferative glomerulonephritis (MPGN) was made (Figure 2B). Retrospective reviews indicated pre-existing proteinuria (2+) with hypoalbuminemia before surgery (38.5 g/L). Postoperatively, the patient received surufatinib for 16 months, with stable disease, and his proteinuria resolved and albumin normalized 3-month post-surgery without any additional interventions.

One month before admission, routine follow-up revealed the onset of NS (4 + proteinuria, 24 h UTP 10.93 g/d, serum albumin 23.2 g/L, LDL-C 8.76 mmol/L) with acute kidney injury (AKI) (serum creatinine 138.2 μmol/L) and radiographic disease progression. Surufatinib was then switched to sandostatin LAR due to suspected nephrotoxicity. Paraneoplastic MPGN was diagnosed based on temporal association, exclusion of other causes, and atypical pathology. Symptomatic treatment was initiated with diuretics, statins, and indobufen due to his intolerance of AECI/ARBs and recent AKI. At discharge, his renal function improved (serum creatinine 86.8 μmol/L) with partial edema resolution.

At the 1-month follow-up, the disease progressed despite sandostatin LAR therapy, prompting transarterial chemoembolization (TACE) and a switch to everolimus. Laboratory results showed decreased proteinuria (2+, 24 h UTP 3.89 g/d) with improved albumin levels (28.5 g/L), lipid profile (LDL-C 2.98 mmol/L), and renal function (serum creatine 71.6 μmol/L, eGFR 107.0 mL/min·1.73m^2^). At 4 months, disease progression continued, complicated by a liver abscess requiring drainage and antibiotic therapy, and NS was exacerbated (3 + proteinuria, serum albumin 23.3 g/L, LDL-C 7.22 mmol/L), leading to everolimus suspension. Partial remission of NS was observed after 6 months (albumin 30.3 g/L without infusion), allowing everolimus resumption. Sustained partial remission was noted at the 1-year follow-up (2 + proteinuria, albumin 34.0 g/L) with stable renal function (serum creatinine 66.3 μmol/L), which persisted over subsequent local follow-up until the last assessment at 1.5 years.

### Case 3

A 72-year-old Chinese man presented with a 5-month history of bilateral lower limb edema. His medical history included atrophic gastritis, treated with rebamipide, and a varicectomy a decade earlier. The patient denied any history of smoking or alcohol consumption. Physical examination revealed mild lower extremity edema. Laboratory findings indicated nephrotic-range proteinuria (24 h UTP 5.28 g/d), microscopic hematuria (4.8 RBCs/HPF), hypoalbuminemia (28.5 g/L), hyperlipidemia (total cholesterol 6.2 mmol/L, LDL-C 4.33 mmol/L), and normal renal function (eGFR 98.0 mL/min·1.73m^2^, CKD-EPI 2009). All serological tests were negative. Abdominal and urinary ultrasonography showed mild fatty liver, prostatic hyperplasia, and vascular atherosclerosis.

**Figure 3:**
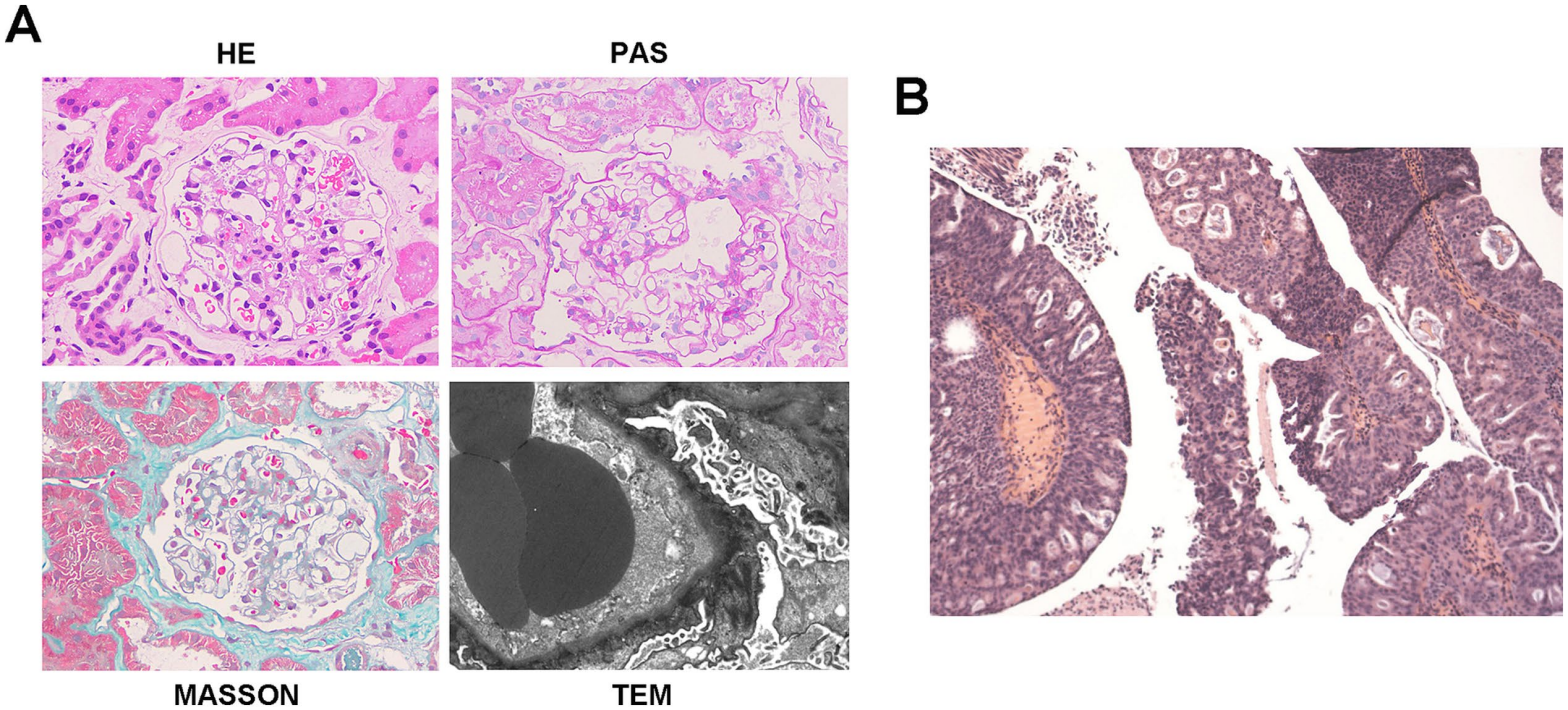
Histopathology of bladder cancer and kidney in case 3. **(A)** Representative of renal pathology of case 3. **(B)** Histopathology shows high-grade papillary urothelial carcinoma. Morphological variant: Focal glandular differentiation. Tumor dimensions: 2.0 × 1.5 × 0.5 cm.

Renal biopsy of 17 glomeruli revealed diffuse capillary wall thickening and podocyte swelling without mesangial proliferation, along with scattered deposits on Masson staining. There was minor tubular atrophy and interstitial fibrosis with mild inflammation. Immunofluorescence showed granular deposits of IgG (IgG1) and light chain deposits along the capillary walls, and PLA2R staining was negative. A diagnosis of atypical membranous nephropathy (MN) or PLA2R-unrelated MN was made (Figure 3A). The patient responded well to diuretics and statins and was discharged on losartan, statins, and indobufen.

At the 4-month follow-up, partial NS remission was observed with reduced proteinuria (24 h UTP 1.56 g/d) and normalized serum albumin (41.6 g/L), though hematuria persisted (40.5 RBCs/HPF). At 5 months, urinary ultrasonography and CT urography showed multiple bladder masses (largest 1.4 × 1.2 cm) and retroperitoneal lymphadenopathy. NS was still in partial remission (24 h UTP 1.44 g/d, serum albumin 39.6 g/L, LDL-C 3.58 mmol/L), with improved urinalysis. The patient underwent transurethral resection of the bladder tumor (TURBT) with postoperative pirarubicin irrigation, which confirmed high-grade papillary urothelial carcinoma with focal adenoid differentiation (Figure 3B). Post-operatively, the patient received Bacillus Calmette–Guerin (BCG) immunotherapy and continued conservative treatment with losartan and dapagliflozin. Three-month post-operatively, near-complete renal remission was achieved, evidenced by minimal proteinuria (24 h UTP 0.62 g/day), normal serum albumin (41.4 g/L), normal lipid levels (LDL-C 1.96 mmol/L), and stable renal function (eGFR 92.49 mL/min/1.73m^2^). This remission was sustained at the 8-month postoperative follow-up, with repeat urinary ultrasonography showing no evidence of recurrence and a further reduction in proteinuria (24 h UTP 0.10 g/day).

## Discussion

Paraneoplastic glomerular diseases refer to glomerular lesions that are indirectly caused by the presence of malignancy through tumor-related mechanisms and which tend to improve following effective treatment of the underlying cancer (5). The clinical diagnosis is often supported by the following characteristics (6, 7): (1) Clinical manifestations of renal injury have a time correlation with tumor (5, 8), especially within 2 years (9); (2) effective treatment of tumor, either surgical or chemical, is the premise for clinical and histologic remission of renal injury (10); (3) deterioration of renal function attributed to tumor relapse (5, 11); and (4) a pathophysiological connection between glomerular lesion and tumorigenesis, namely tumor antigens or antibody components detected by pathological examination (6). Among these, MN represents the most frequently reported histologic pattern, although other pathological alterations may occur (5, 12, 13). Importantly, there are currently no established consensus guidelines for the management of both cancer and paraneoplastic glomerulopathy (14, 15). In this context, we present three rare instances of solid malignancy-associated NS, including ovarian cancer, pancreatic NET, and bladder carcinoma, which are highly suggestive of a paraneoplastic etiology. A paramount and unifying observation across all cases is the achievement of complete or partial NS remission solely through antitumor and supportive therapies, without the use of corticosteroids or immunosuppressive agents. This finding accentuates a critical role of underlying malignancy in the pathogenesis of glomerulopathy in these patients and underscores the therapeutic primacy of oncological control. Clinical characteristics, management, and outcomes of three presenting cases are summarized in Table 1.

**Table 1:** Clinical characteristics, management, and outcomes of the three presenting cases with PNS.

Indicators	Case 1	Case 2	Case 3
Demographics			
Age (years)/Sex	71/F	57/M	72/M
Malignancy			
Tumor type	Ovarian cancer	Pancreatic NET	Bladder cancer
Histology/Stage	Stage IIIC high-grade serous ovarian carcinoma	G2	High-grade papillary urothelial carcinoma
Renal involvement			
Pathology	NA	MPGN	MN
24 h UTP (g/d)	12.80	10.93	5.28
Serum Albumin (g/L)	19.30	23.20	28.50
Treatment			
Antitumor therapy	Cytoreductive surgery, Paclitaxel/Carboplatin, Bevacizumab	Surufatinib, Sandostatin LAR, TACE, Everolimus	TURBT, Pirarubicin irrigation, BCG immunotherapy
Renal therapy	Supportive care, Irbesartan	Supportive care	Losartan, Dapagliflozin
Steroids/Immunosuppression	None	None	None
Outcome			
Renal response	Complete remission	Partial remission	Complete remission
Oncologic response	Progressive disease	Progressive disease	Stable disease

Ovarian cancer, one of the most common gynecologic malignancies, frequently presents with non-specific symptoms in its early stages (16, 17). Its association with paraneoplastic glomerulopathy remains rare, with only 20 documented cases to date (including 5 benign teratomas) (Table 2) (18–33). Notably, 70% (14/20) of these patients achieved renal remission during follow-up, despite approximately half receiving steroids or immunosuppressive therapy, suggesting a favorable renal prognosis in ovarian cancer-associated PNS, although oncologic outcomes were frequently unreported. Recent studies have also indicated associations between gynecological neoplasms and glomerular lesions, although the histological characteristics remain undefined (4). In our first case, the temporal dynamics between tumor activity and renal response were particularly revealing. Although a definitive histological diagnosis is lacking, complete and sustained NS remission was induced following cytoreductive surgery and chemotherapy, providing strong clinical evidence for a paraneoplastic etiology. Although the patient had been on long-term irbesartan therapy prior to the onset of NS, and spontaneous remission could not be entirely ruled out, the immediate response to tumor debulking remains the most plausible explanation and underscores the value of steroid-sparing approaches in such scenarios. Another important point is the potential nephrotoxicity of oncological therapies, such as bevacizumab, used in this case. Bevacizumab is a monoclonal antibody targeting vascular endothelial growth factor (VEGF-A) and is known to be associated with proteinuria and other glomerular lesions, such as glomerular microangiopathy (34, 35). Critically, the temporal sequence of events in this patient, which showed significant renal improvement after tumor-directed therapy but persisted despite the administration of bevacizumab, supports the primacy of a paraneoplastic mechanism–rather than drug-induced nephrotoxicity—is the primary cause. Notably, the renal remission in this case proved remarkably resilient, persisting even upon subsequent oncologic progression, a phenomenon rarely documented in prior ovarian cancer-associated PNS cases. This suggests that initial reduction in tumor burden might disrupt paraneoplastic pathways sufficiently to induce prolonged stabilization of glomerular permeability, independent of later tumor progression, which is consistent with previous findings that glomerular lesions may be indirectly related to tumor burden, invasion, or metastasis (5, 6).

**Table 2:** Summary of reported PNS associated with ovarian cancer.

Cases	Age	Clinical onset of renal lesion	Discovery of neoplasm	Renal lesion	Type of cancer	Treatment	Outcome at last follow up	
				Pathology	Histology of ovarian cancer		Renal outcome	Tumor outcome
#1 (20)	65	1987	1987	NA	Metastatic adenocarcinoma from an ovarian primary	Surgery and chemotherapy	Partial remission	Unknown
#2 (24)	68	April 2000	May 2000	NA	Ovarian adenocarcinoma	Surgery and chemotherapy	Partial remission	Death
#3 (29)	73	October 2008	October 2008	MCD	Papillary serous adenocarcinoma	Steroids and immunosuppressive therapy Chemotherapy with paclitaxel and carboplatin and tumor-debulking surgery	No response Complete remission	Unknown
#4 (30)	6	NA	NA	MN	Sertoli-Leydig ovarian tumor	Surgery and chemotherapy	Remission	Unknown
#5 (67)	65	6 months before tumor	NA	MN	NA	Chemotherapy	Remission	Normalize CA125
#6 (25)	59	October 2000	May 2001	MN	Ovarian carcinoma	Chemotherapy	Complete remission	Complete remission
#7 (21)	7	March 1979	December 1979	MN	Teratoma	Prednisone and surgery	Complete remission	Unknown
#8 (33)	NA	1988	NA	MN	Ovarian adenocarcinoma	Steroids and surgery	Remission	Unknown
#9 (26)	55	December 2002	December 2002	MCD	Papillary serous carcinoma	Prednisolone and adjuvant chemotherapy	Complete remission	Unknown
#10 (27)	65	January 2007	November 2004	MN	Ovarian clear cell carcinoma	Surgery, steroid and cyclophosphamide	Remission	Alive
#11 (31)	55	December 2008	December 2008	MCD	Teratoma	Surgery combined with corticosteroids	Complete remission	Unknown
#12 (19)	36	2013	NA	MCD	Teratoma	Surgery and prednisone	Remission	Unknown
#13 (32)	46	2019	NA	MN	Serous cyst adenofibroma	Steroids and surgery	Partial remission	Unknown
#14 (28)	16	NA	25 days after NS	MPGN	Teratoma	Prednisone, surgery and Cyclosporine A	Complete remission	Unknown
#15 (18)	65	April 1960	November 1961	MN	Ovarian adenocarcinoma	Surgery	Unknown	Death
#16 (22)	64	October 1992	October 1993	AL	Ovarian carcinoma	Surgery	Renal failure	Death
#17 (23)		February 1992	October 1994	MPGN	Ovarian endodermal sinus tumor	Prednisone and ACEI; surgery and chemotherapy	Renal failure	Unknown
#18/19 (68)	59/72	NA	NA	MCD/MCD	NA	Surgery	One with renal failure for, the other unknown	Unknown
#20 (18)	28	After tumor removal	NA	MN	Ovarian dermoid cyst	Steroid	No response	Unknown

In this case, the initial parallel decline of both CA125 and HE4 with NS remission, followed by their subsequent dissociation (isolated HE4 elevation during cancer progression without NS relapse), revealed a fascinating nuance not previously emphasized in the literature. This dissociation may be clinically significant, since HE4 has been shown to possess higher specificity for ovarian malignancy than CA125 (36, 37), and its elevation has been observed in chronic kidney disease (CKD) independent of renal function (38, 39). Therefore, the isolated HE4 increase likely reflected genuine tumor progression, whereas the sustained NS remission suggested a decoupling of the paraneoplastic mechanism. This observation suggests complex biomarker interactions in PNS that merit further investigation.

Neuroendocrine neoplasms (NENs) represent a diverse group of tumors predominantly originating from gastroenteropancreatic (GEP) tissues (40, 41). The majority are indolent neuroendocrine tumors (NETs), while approximately 10–20% are neuroendocrine carcinomas (NECs) with rapid disease progression (41, 42). To date, only two cases of NEN-associated PNS have been reported worldwide, one including a case of pancreatic NEC (43), and another a pancreatic NET (Table 3) (44). Our study presents the third global case and the first Asian case of pancreatic NEN-related PNS. Of particular interest is the temporal pattern of the paraneoplastic glomerulopathy, occurring before the administration of sunitinib and remitting during postoperative surufatinib therapy, and recurring with tumor progression. This pattern strongly suggests that tumor burden, rather than drug toxicity (45, 46), serves as the primary driver of renal injury, similar to our first case. More notably, subsequent tumor progression under everolimus therapy was paradoxically associated with partial NS remission, indicating a complex and non-linear relationship between tumor burden and paraneoplastic glomerulopathy. This observation also suggests a potential nephroprotective role for everolimus through mTORC1 inhibition, as demonstrated in previous studies (36, 37), adding a novel therapeutic dimension to the management of such complex cases that has not been adequately described in the existing literature.

**Table 3:** Summary of PNS associated with NEN and bladder tumor.

Case	Age	Sex	Renal lesion	Type of cancer	Treatment	Outcome	
			Pathology	Histology of NEN		Renal outcome	Tumor outcome
#1 (43)	69	Male	MN	High-grade neuroendocrine carcinoma (pT3N0)	Surgery	AKI	Unknown
#2 (44)	57	Female	MN	Non-malignant NET	Surgery, Steroids, Telmisartan	Partial remission	Stable disease
			Pathology	Histology of bladder cancer		Renal outcome	Tumor outcome
#1 (47)	NA	NA	NA	NA	NA	NA	NA
#2 (53)	66	Female	MCD	Bladder transitional cell carcinoma	Surgery	Complete remission	Unknown
#3 (48)	50	Male	MPGN	Bladder transitional cell carcinoma	Surgery	Complete remission	Unknown
#4 (49)	75	Male	MN	Bladder transitional cell carcinoma	Surgery	Remission	Unknown
#5 (54)	54	Male	MN	Low-grade transitional cell carcinoma	Surgery, BCG injection, Steroids	Remission	Remission
#6 (50)	68	Male	MN	THSD7A-positive bladder cancer	Steroid; Surgery	No response; Remission	Unknown
#7 (51)	57	Male	Renal amyloidosis	Bladder small cell carcinoma	Unknown	Unknown	Unknown
#8 (52)	76	Male	NA	Bladder urothelial carcinoma	Surgery	Complete remission	Death

In the literature, only eight cases of bladder tumor-related paraneoplastic NS have been reported (47–54), with four cases demonstrating partial or complete NS remission after surgical treatment. Corticosteroid therapy failed to improve proteinuria in two of these cases (49, 50), contrasting with the more favorable renal outcomes observed in ovarian cancer-associated PNS. Our third case of bladder cancer-associated MN provides critical new insights into its diagnostic and therapeutic challenges. Although the pathological pattern itself may not be unusual, the clinical presentation and course were highly instructive, expanding upon the eight previously reported cases of bladder tumor-related PNS (47–54). Different from prior cases (Table 3), in our patient, the manifestation of NS preceded bladder malignancy detection by several months. Significantly, partial remission was achieved with losartan monotherapy even before tumor detection, and this remission was further accelerated following surgical resection. This might be attributed to diagnostic limitations, since initial urological screening with ultrasonography revealed no masses at the time of NS diagnosis, while sensitive methods, including CT urography or contrast-enhanced ultrasound, are not routinely applied for scanning NS patients in the clinical setting, even in the elderly. Furthermore, while malignancy-associated MN is associated with other antigens such as THSD7A (13, 55), these biomarker assays are not yet widely integrated into routine clinical practice in our institution, due to cost constraints. Importantly, the observed treatment response in the third case indicates a dual potential mechanism: first, the reduction in tumor burden removes the antigenic stimulus; second, angiotensin receptor blockers (ARBs) may exert nephroprotective and potential antitumor effects by inhibiting the renin–angiotensin–aldosterone system (RAAS), which has been implicated in tumorigenesis via signaling pathways including Ras/RAF/MAPK/ERK, PI3K/AKT/mTOR, and Wnt/*β*-catenin (56–61). Therefore, this case highlights the importance of maintaining a high index of suspicion for occult malignancy in patients with NS, even after an initially negative urological evaluation, and the importance of scheduled monitoring, even after treatment initiation.

Current understanding of paraneoplastic glomerulopathy pathogenesis remains incomplete despite several proposed molecular mechanisms (62–66). Our cases contribute to this understanding by demonstrating that: (1) the relationship between tumor burden and glomerulopathy is not always linear and may exhibit complex temporal dynamics; (2) different cancer types may use distinct pathophysiological pathways to induce glomerular injury, which may be reflected in variations of specific biomarkers; (3) non-immunosuppressive treatments, including both RAAS inhibition and targeted antitumor agents, can be effective while potentially minimizing side effects; and (4) certain antineoplastic agents may possess underrecognized nephroprotective effects in PNS. Future research should prioritize the identification of novel biomarkers through the analysis of renal biopsy specimens from cancer patients with glomerular involvement, combined with functional validation in reliable *in vitro* and *in vivo* models. Large-scale cohort studies are needed to identify epidemiological characteristics, including the influence of race, ethnicity, age, and gender, as well as temporal relationships between tumor diagnosis and renal manifestations. Such investigations would advance our understanding of disease mechanisms and contribute to the establishment of more precise diagnostic criteria and prognostic indicators for this complex paraneoplastic phenomenon.

## Data Availability

The raw data supporting the conclusions of this article will be made available by the authors, without undue reservation.

## References

[1] Galloway J, Remarks ON HODGKIN'S DISEASE. Br Med J, (1922). 2: p. 1201–1208.2. 20770949 10.1136/bmj.2.3234.1201 PMC2417333

[2] Puolijoki H Mustonen J Pettersson E Pasternack A Lahdensuo A. Proteinuria and haematuria are frequently present in patients with lung cancer. Nephrol Dial Transplant. (1989) 4:947–50. doi: 10.1093/ndt/4.11.947, PMID: 2516885

[3] Sawyer N Wadsworth J Wijnen M Gabriel R. Prevalence, concentration, and prognostic importance of proteinuria in patients with malignancies. Br Med J (Clin Res Ed). (1988) 296:1295–8. doi: 10.1136/bmj.296.6632.1295, PMID: 3133055 PMC2545767

[4] Laferreira MS Kirsztajn GM. Potentially paraneoplastic glomerulopathies in a Brazilian cohort: a retrospective analysis. J Bras Nefrol. (2025) 47:e20240131. doi: 10.1590/2175-8239-jbn-2024-0131en, PMID: 39878345 PMC11781679

[5] Lien YH Lai LW. Pathogenesis, diagnosis and management of paraneoplastic glomerulonephritis. Nat Rev Nephrol. (2011) 7:85–95. doi: 10.1038/nrneph.2010.171, PMID: 21151207 PMC3058941

[6] Ronco PM. Paraneoplastic glomerulopathies: new insights into an old entity. Kidney Int. (1999) 56:355–77. doi: 10.1046/j.1523-1755.1999.00548.x, PMID: 10411717

[7] Bacchetta J Juillard L Cochat P Droz JP. Paraneoplastic glomerular diseases and malignancies. Crit Rev Oncol Hematol. (2009) 70:39–58. doi: 10.1016/j.critrevonc.2008.08.003, PMID: 18790651

[8] Pani A Porta C Cosmai L Melis P Floris M Piras D. Glomerular diseases and cancer: evaluation of underlying malignancy. J Nephrol. (2016) 29:143–52. doi: 10.1007/s40620-015-0234-9, PMID: 26498294 PMC4792341

[9] Caza TN Hassen SI Dvanajscak Z Kuperman M Edmondson R Herzog C. NELL1 is a target antigen in malignancy-associated membranous nephropathy. Kidney Int. (2021) 99:967–76. doi: 10.1016/j.kint.2020.07.039, PMID: 32828756 PMC7895854

[10] Lefaucheur C Stengel B Nochy D Martel P Hill GS Jacquot C. Membranous nephropathy and cancer: epidemiologic evidence and determinants of high-risk cancer association. Kidney Int. (2006) 70:1510–7. doi: 10.1038/sj.ki.5001790, PMID: 16941021

[11] Cambier JF Ronco P. Onco-nephrology: glomerular diseases with cancer. Clin J Am Soc Nephrol. (2012) 7:1701–12. doi: 10.2215/CJN.03770412, PMID: 22904123

[12] Royal V Leung N Karam S Bridoux F Nasr SH. Paraneoplastic Glomerulopathies: mechanistic and pathogenic insights. Am J Nephrol. (2025) 23:1–13. doi: 10.1159/000546050, PMID: 40267904

[13] Murt A Berke I Bruchfeld A Caravaca-Fontán F Floege J Frangou E. Malignancies and glomerulonephritis: when to suspect and when to screen? Clin Kidney J. (2025) 18:sfaf101. doi: 10.1093/ckj/sfaf101, PMID: 40352576 PMC12062743

[14] Porta C Bamias A Danesh FR Dębska-Ślizień A Gallieni M Gertz MA. KDIGO controversies conference on onco-nephrology: understanding kidney impairment and solid-organ malignancies, and managing kidney cancer. Kidney Int. (2020) 98:1108–19. doi: 10.1016/j.kint.2020.06.046, PMID: 33126977

[15] Cozzo D Orlando F Bruno M Ogna A Forni Ogna V. Minimal change glomerular disease associated with solid neoplasms: a systematic review. J Nephrol. (2024) 38:343–52. doi: 10.1007/s40620-024-02084-6, PMID: 39352607 PMC11961479

[16] Siegel RL Miller KD Jemal A. Cancer statistics, 2018. CA Cancer J Clin. (2018) 68:7–30. doi: 10.3322/caac.21442 29313949

[17] Beck LH Jr. Membranous nephropathy and malignancy. Semin Nephrol. (2010) 30:635–44. doi: 10.1016/j.semnephrol.2010.09.011 21146128

[18] Lee JC Yamauchi H Hopper J Jr. The association of cancer and the nephrotic syndrome. Ann Intern Med. (1966) 64:41–51. doi: 10.7326/0003-4819-64-1-41, PMID: 5900782

[19] Jeroudi A Kadikoy H Gaber L Ramanathan V Frome A Anwar N. Profound nephrotic syndrome in a patient with ovarian teratoma. Saudi J Kidney Dis Transpl. (2013) 24:777–82. doi: 10.4103/1319-2442.113883, PMID: 23816730

[20] Hoyt RE Hamilton JF. Ovarian cancer associated with the nephrotic syndrome. Obstet Gynecol. (1987) 70:513–4. PMID: 3627617

[21] Beauvais P Vaudour G Gibod LB Levy M. Membranous nephropathy associated with ovarian tumour in a young girl: recovery after removal. Eur J Pediatr. (1989) 148:624–5. doi: 10.1007/BF00441515 2663514

[22] Fernandez-Miranda C Mateo S Gonzalez-Gomez C Ballestin C. Systemic amyloidosis and ovarian carcinoma. Postgrad Med J. (1994) 70:505–6. doi: 10.1136/pgmj.70.825.505, PMID: 7937430 PMC2397667

[23] Salazar-Exaire D Rodríguez A Galindo-Rujana ME Briones JC Arenas-Osuna J Rocha LM. Membranoproliferative glomerulonephritis associated with a mixed-cell germinal ovary tumor. Am J Nephrol. (2001) 21:51–4. doi: 10.1159/000046219, PMID: 11275633

[24] Forgy AP Ewing TL Flaningam J. Two paraneoplastic syndromes in a patient with ovarian cancer: nephrotic syndrome and paraneoplastic cerebellar degeneration. Gynecol Oncol. (2001) 80:96–8. doi: 10.1006/gyno.2000.6029, PMID: 11136578

[25] Kim YT Rha SY Shim CY Sohn JH Kim C Yu NC. A case of paraneoplastic nephrotic syndrome in a patient with ovarian carcinoma. Yonsei Med J. (2003) 44:539–43. doi: 10.3349/ymj.2003.44.3.539, PMID: 12833596

[26] Ryu DR Yoo TH Kim YT Jeong HJ Cho NH Kang SW. Minimal change disease in a patient with ovarian papillary serous carcinoma. Gynecol Oncol. (2004) 93:554–6. doi: 10.1016/j.ygyno.2004.02.022, PMID: 15099980

[27] Ata A Gürses I Kıykım A Arıcan A. Nephrotic syndrome associated with gemcitabine use in a patient with ovarian cancer. Am J Case Rep. (2012) 13:268–70. doi: 10.12659/AJCR.883583, PMID: 23569546 PMC3614261

[28] Kilis-Pstrusinska K Szajerka U Zwolinska D. Unspecific increase of tumor markers in a girl with nephrotic syndrome and ovarian teratoma. Ren Fail. (2013) 35:654–6. doi: 10.3109/0886022X.2013.780614, PMID: 23560847

[29] González-Fontal GR Restrepo JG Henao-Martínez AF. Minimal-change disease as a paraneoplastic syndrome in a patient with ovarian carcinoma. NDT Plus. (2011) 4:427–9. doi: 10.1093/ndtplus/sfr106 25984215 PMC4421649

[30] Chen A Vander Lugt M McAllister-Lucas LM Killen PD Blatt NB. An unusual case of membranous nephropathy associated with an ovarian tumor. Pediatr Nephrol. (2011) 26:2249–51. doi: 10.1007/s00467-011-1994-7, PMID: 21892796

[31] Benabdellah N Izzedine H Bentata Y Haddiya I. Ovarian tumor and glomerulopathies: case report and review of the literature. Pan Afr Med J. (2019) 34:75. doi: 10.11604/pamj.2019.34.75.6008 31819791 PMC6884736

[32] Ana Stojanoska NG Simona S-G Svetlana Pavleska-Kuzmanovska LT Severova G Rambabova-Bushljetic I Pushevski V. Membranous nephropathy as a paraneoplastic syndrome in a patient with ovarian serous Cystadenofibroma - case report. Bantao Journal. (2019) 17:108–10.

[33] Torres VE JV Donadio Jr., Cical aspects of the nephrotic syndrome in systemic diseases. New York, Dekker, (1988). in Cameron SI Glassock RJ(eds): Nephrotic syndrome, ed 1.: 555–651.

[34] Onteddu NK Mudupula Vemula SS Areddy VR Onteddu J Mabbu T. Bevacizumab-induced nephropathy presenting as crescentic Glomerulopathy. Cureus. (2023) 15:e48787. doi: 10.7759/cureus.48787, PMID: 38098914 PMC10720257

[35] Jiang R Yu RZ Yang HF Wang LX Lin JJ. Bevacizumab-associated glomerular microangiopathy: a case report and literature review. BMC Nephrol. (2025) 26:445. doi: 10.1186/s12882-025-04385-9, PMID: 40783678 PMC12335079

[36] Moore RG Brown AK Miller MC Skates S Allard WJ Verch T. The use of multiple novel tumor biomarkers for the detection of ovarian carcinoma in patients with a pelvic mass. Gynecol Oncol. (2008) 108:402–8. doi: 10.1016/j.ygyno.2007.10.017, PMID: 18061248

[37] Zhang M Zhang Y Liu G Ge L. A new risk algorithm combining D-dimer and HE4 differentiates borderline tumor from patients with ovarian tumor. Transl Cancer Res. (2025) 14:93–101. doi: 10.21037/tcr-24-1276, PMID: 39974418 PMC11833360

[38] Bolstad N Øijordsbakken M Nustad K Bjerner J. Human epididymis protein 4 reference limits and natural variation in a Nordic reference population. Tumour Biol. (2012) 33:141–8. doi: 10.1007/s13277-011-0256-4, PMID: 22105734 PMC3235278

[39] Yuan T Li Y. Human epididymis protein 4 as a potential biomarker of chronic kidney disease in female patients with Normal ovarian function. Lab Med. (2017) 48:238–43. doi: 10.1093/labmed/lmx036, PMID: 28934517

[40] Dasari A Shen C Halperin D Zhao B Zhou S Xu Y. Trends in the incidence, prevalence, and survival outcomes in patients with neuroendocrine tumors in the United States. JAMA Oncol. (2017) 3:1335–42. doi: 10.1001/jamaoncol.2017.0589, PMID: 28448665 PMC5824320

[41] Das S Dasari A. Epidemiology, incidence, and prevalence of neuroendocrine neoplasms: are there global differences? Curr Oncol Rep. (2021) 23:43. doi: 10.1007/s11912-021-01029-7, PMID: 33719003 PMC8118193

[42] Sorbye H Strosberg J Baudin E Klimstra DS Yao JC. Gastroenteropancreatic high-grade neuroendocrine carcinoma. Cancer. (2014) 120:2814–23. doi: 10.1002/cncr.28721 24771552

[43] Gleeson FC Brown CM Herrera Hernandez LP. A pancreatic mass and bilateral pitting pedal edema: nothing is ever what it seems. Clin Gastroenterol Hepatol. (2012) 10:e3. doi: 10.1016/j.cgh.2011.08.018, PMID: 21893133

[44] Forslund T Kellokumpu I Elomaa E Arola J Nuorva K. Remission of membranous glomerulonephritis after pancreatectomy for pancreatic neuroendocrine neoplasm - a rare coincidence. Clin Nephrol. (2011) 75:42–6. doi: 10.5414/CNP75042 21269593

[45] Singh L Matassa D Li S. A case of acute kidney injury, proteinuria, and thrombotic Microangiopathy associated with Sunitinib therapy in metastatic pancreatic neuroendocrine tumor. Cureus. (2024) 16:e56660. doi: 10.7759/cureus.56660, PMID: 38646245 PMC11032219

[46] Khan A Consing Gangelhoff M Moubarak S Herrmann S Nooruddin K Alexander M. Sunitinib induced glomerular thrombotic microangiopathy in a patient with refractory pancreatic neuroendocrine tumour. J Clin Pathol. (2024) 78:357–8. doi: 10.1136/jcp-2024-209851, PMID: 39674583 PMC12015045

[47] Fukushi Y Sugita A Ozu K. A case of bladder tumor associated with nephrotic syndrome Fukuoka: Nishinihon J. Urol (1978) 40:89–93.

[48] Reshi AR Mir SA Gangoo AA Shah S Banday K. Nephrotic syndrome associated with transitional cell carcinoma of urinary bladder. Scand J Urol Nephrol. (1997) 31:295–6. doi: 10.3109/00365599709070351 9249896

[49] Fabbian F Rizzioli E Catalano C. Membranous glomerulonephritis and transitional cell carcinoma, improved proteinuria after each tumor resection. G Ital Nefrol. (2003) 20:65–8. 12647289

[50] Matsumoto A Matsui I Mano K Mizuno H Katsuma Y Yasuda S. Recurrent membranous nephropathy with a possible alteration in the etiology: a case report. BMC Nephrol. (2021) 22:253. doi: 10.1186/s12882-021-02457-0, PMID: 34229600 PMC8258946

[51] Kanat O Evrensel T Filiz G Usta M Baskan E Dilek K. Systemic AA amyloidosis and nephrotic syndrome associated with small cell carcinoma of the bladder. Nephrol Dial Transplant. (2003) 18:2453-a–4. doi: 10.1093/ndt/gfg391 14551390

[52] Mizusawa H Mimura Y Utazu H Maejima T. Muscle invasive urinary bladder urothelial carcinoma presenting with secondary nephrotic symptoms. IJU Case Rep. (2021) 4:314–7. doi: 10.1002/iju5.12335, PMID: 34497993 PMC8413222

[53] Rapoport J Kuperman O Gopas Y Maor E Eyal A Mostovslavski M. Nephrotic syndrome associated with transitional cell carcinoma of the bladder. Nephron. (1989) 52:36–9. doi: 10.1159/000185579, PMID: 2651948

[54] Singh NP Prakash A Kubba S Ganguli A Agarwal SK Dinda AK. Nephrotic syndrome as a complication of intravesical BCG treatment of transitional cell carcinoma of urinary bladder. Ren Fail. (2007) 29:227–9. doi: 10.1080/08860220601098961, PMID: 17365941

[55] Hoxha E Beck LH Wiech T Tomas NM Probst C Mindorf S. An indirect immunofluorescence method facilitates detection of thrombospondin type 1 domain-containing 7A-specific antibodies in membranous nephropathy. J Am Soc Nephrol. (2017) 28:520–31. doi: 10.1681/ASN.2016010050, PMID: 27436855 PMC5280014

[56] Pawlonka J Buchalska B Buczma K Borzuta H Kamińska K Cudnoch-Jędrzejewska A. Targeting the renin-angiotensin-aldosterone system (RAAS) for cardiovascular protection and enhanced oncological outcomes: review. Curr Treat Options in Oncol. (2024) 25:1406–27. doi: 10.1007/s11864-024-01270-9, PMID: 39422794 PMC11541340

[57] Forrester SJ Booz GW Sigmund CD Coffman TM Kawai T Rizzo V. Angiotensin II signal transduction: an update on mechanisms of physiology and pathophysiology. Physiol Rev. (2018) 98:1627–738. doi: 10.1152/physrev.00038.2017, PMID: 29873596 PMC6335102

[58] Siljee S Pilkington T Brasch HD Bockett N Patel J Paterson E. Cancer stem cells in head and neck metastatic malignant melanoma express components of the renin-angiotensin system. Life (Basel). (2020) 10:268. doi: 10.3390/life10110268, PMID: 33147716 PMC7694034

[59] Kilmister EJ Tan ST. Cancer stem cells and the renin-angiotensin system in the tumor microenvironment of melanoma: implications on current therapies. Int J Mol Sci. (2025) 26:1389. doi: 10.3390/ijms26031389, PMID: 39941158 PMC11818896

[60] Wang J Nishiyama A Matsuyama M Wang Z Yuan Y. The (pro)renin receptor: a novel biomarker and potential therapeutic target for various cancers. Cell Commun Signal. (2020) 18:39. doi: 10.1186/s12964-020-0531-3, PMID: 32143717 PMC7060546

[61] Sever N Yunusov E Çelebi A Yaşar A Majidova N Kocaaslan E. Impact of renin angiotensin system inhibitors on survival of patients with metastatic non-small cell lung cancer. Ann Saudi Med. (2025) 45:18–24. doi: 10.5144/0256-4947.2025.18, PMID: 39929787 PMC11810880

[62] Bertelli R Bonanni A Caridi G Canepa A Ghiggeri GM. Molecular and cellular mechanisms for proteinuria in minimal change disease. Front Med (Lausanne). (2018) 5:170. doi: 10.3389/fmed.2018.00170, PMID: 29942802 PMC6004767

[63] Stuchlova Horynova M Raška M Clausen H Novak J. Aberrant O-glycosylation and anti-glycan antibodies in an autoimmune disease IgA nephropathy and breast adenocarcinoma. Cell Mol Life Sci. (2013) 70:829–39. doi: 10.1007/s00018-012-1082-6, PMID: 22864623 PMC3745718

[64] McAdoo SP Pusey CD. Antiglomerular Basement Membrane Disease. Semin Respir Crit Care Med. (2018) 39:494–503. doi: 10.1055/s-0038-1669413, PMID: 30404116

[65] Li JN Cui Z Wang J Hu SY Jia XY Guan Z. Autoantibodies against linear epitopes of myeloperoxidase in anti-glomerular basement membrane disease. Clin J Am Soc Nephrol. (2016) 11:568–75. doi: 10.2215/CJN.05270515, PMID: 26813562 PMC4822661

[66] Koyama A Fujisaki M Kobayashi M Igarashi M Narita M. A glomerular permeability factor produced by human T cell hybridomas. Kidney Int. (1991) 40:453–60. doi: 10.1038/ki.1991.232 1787645

[67] Topalak O Saygili U Soyturk M Karaca N Batur Y Uslu T. Serum, pleural effusion, and ascites CA-125 levels in ovarian cancer and nonovarian benign and malignant diseases: a comparative study. Gynecol Oncol. (2002) 85:108–13. doi: 10.1006/gyno.2001.6575, PMID: 11925128

[68] Lionaki S Marinaki S Panagiotellis K Tsoumbou I Liapis G Vlahadami I. Glomerular diseases associated with malignancies: histopathological pattern and association with circulating autoantibodies. Antibodies. (2020) 9:18. doi: 10.3390/antib9020018, PMID: 32466285 PMC7345950

